# Obituary

**Published:** 1863-12

**Authors:** 


					﻿DIED— At Piqua, 0., Nov. 19th, 1863, Fred. B. White,
dental student to Drs. Brown & Dills—aged 27 years.
I will not presume to write, here, the obitue^v of Fred-
erick B. White, due one older in the profession, or due him-
self in another place; but I will be permitted to write of
him as of one promising much for the profession as a type
of a desirable class of dental students, and as a companion of
one who feels his loss too deeply to refrain from any expres-
sion possible of his noble life, or lamented death.
Mr. White will be remembered by those connected with
the Ohio Dental College during the years 1856, 1857 and
1858, as a friend and room-mate of the writer,—of tall,
unusually commanding figure, combined with an affability
irresistible in its tendency to form the acquaintance and
friendship of all with whom he came in contact. A son of
the late Dr. White—for twenty years President of the Wa-
bash College, Crawfordsville, Ind. He enjoyed the advan-
tages of a collegiate education, as well as those of a high
social association. These general advantages were fully im-
proved by the individual disposition. Never was culture be-
stowed upon more fruitful ground, nor labor more success-
fully spent in the formation of character ; never was a young
man’s character more steadfast and elevated. This was his
characteristic,—abounding and unyielding in habits of virtue
and religion. Many and many number the nights I have
seen him retire, yet never one without reading his Bible.
And such habits were pleasingly blended with those natural
to a highly social life. A serious invalid for four- years
prior to his death, most remarkably did he mingle, as it were,
life and death,—walking hand in band with his sable antago-
nist, as with an acquaintance neither dreaded nor desired.
Ever noble and good, he was now afflicted, yet patient; suf-
fering, yet resigned—perfectly resigned—approaching his
grave
“Like one that draws the drapery of his couch
About him, and lies down to pleasant dreams.”
But Oh ! how hard for the living to resign “dust to dust”
—the once noble breathing friend, to cold inanimate clay !
no more to hear the accustomed footfall: no more to see
the familiar face and gesture ; or receive the greetings of
his friendship, or the deeds of his kindness. Ah! no more
is it for the accustomed members of our dental laboratory to
gather round a common work-table, and make the sweat of
the brow pleasant by association, and the laugh merrier by
the many, and the sigh softer by the mutual sympathy !
Yet the bereavement of a few, by a sacred tie severed, is
not to be obtruded on the many, only so far as it may re-
mind us all, that
“In the midst of life we are in death;”
and as we would appreciate life, and prepare for death, we
would study, cultivate, and discharge our duties to the liv-
ing—worshiping the relationships of life—as affording our
highest duties, and purest joys.	C. C. Dalls.
Piqua, 0., Dec. 9, 1863.
				

